# Utility of the Bacteriophage RB69 Polymerase gp43 as a Surrogate Enzyme for Herpesvirus Orthologs 

**DOI:** 10.3390/v5010054

**Published:** 2013-01-08

**Authors:** Nicholas Bennett, Matthias Götte

**Affiliations:** 1 Department of Microbiology and Immunology, McGill University, 3775 University Street, Montreal, Quebec H3A 2B4, Canada; 2 Department of Biochemistry, McGill University, 3655 Sir William Osler Promenade, Montreal, Quebec H3G1Y6, Canada; 3 Department of Medicine, Division of Experimental Medicine, McGill University, 1110 Pine Avenue West, Montreal, Quebec H3A 1A3, Canada

**Keywords:** DNA polymerase, T4 DNA polymerase, gp43, *herpesviridae*, UL30, UL54, HSV1, HCMV, RB69 DNA polymerase

## Abstract

Viral polymerases are important targets in drug discovery and development efforts. Most antiviral compounds that are currently approved for treatment of infection with members of the *herpesviridae* family were shown to inhibit the viral DNA polymerase. However, biochemical studies that shed light on mechanisms of drug action and resistance are hampered primarily due to technical problems associated with enzyme expression and purification. In contrast, the orthologous bacteriophage RB69 polymerase gp43 has been crystallized in various forms and therefore serves as a model system that provides a better understanding of structure–function relationships of polymerases that belong the type B family. This review aims to discuss strengths, limitations, and opportunities of the phage surrogate with emphasis placed on its utility in the discovery and development of anti-herpetic drugs.

## Nomenclature

HSV1: Herpes Simplex Virus 1
HSV2: Herpes Simplex Virus 2
VZV: Varicella Zoster Virus
EBV: Epstein–Barr Virus
HCMV: Human cytomegalovirus
HHV6: Human Herpesvirus 6
HHV7: Human Herpesvirus 7
KSHV: Kaposi’s sarcoma-associated herpesvirus
PFA: Phosphonoformic acid
PAA: Phosphonoacetic acid
ACV: Acyclovir
GCV: Ganciclovir
CDV: Cidofovir

## 1. Introduction

The eukaryotic viruses of the *herpesviridae* family are important human pathogens. In all, there are eight different human herpesviruses; Herpes simplex virus 1 and 2 (HSV1: HHV1 and HSV2: HHV2), varicella zoster virus (VZV: HHV3), Epstein-Barr virus (EBV: HHV4), Human cytomegalovirus (HCMV: HHV5), Human Herpes virus 6 and 7 (HHV6 and HHV7) and Kaposi’s sarcoma-associated herpesvirus (KSHV, HHV8). Human herpesviruses cause a spectrum of diseases ranging from relatively benign cutaneous lesions to serious conditions like encephalitis and cancer. Viruses that belong to the *herpesviridae* family are characterized by their ability to establish lifelong, latent infections. Thus, a substantial proportion of the global population is seropositive for one or more herpesviridae viruses. Although individuals with a functioning immune system can generally keep the virus suppressed, the ability to form latent infections, and the fact that the virus is widespread in the human population means that herpesvirus reactivation is a major source of disease and morbidity in immunocompromised individuals. 

The majority of approved antiviral drugs have been shown to inhibit the herpesvirus-specific DNA polymerase, reducing viral DNA replication, and, in turn, viral load [1]. However, although herpesvirus polymerases are all structurally related, they are not highly homologues. As a consequence, most drugs do not show broad antiviral activities against the various members of the *herpesviridae*. The nucleoside analog acyclovir (ACV) and its pro-drug valacyclovir are utilized to treat infection with HSV1, HSV2 or VZV, while the nucleotide analog ganciclovir (GCV) (or valganciclovir) and cidofovir (CDV) are approved to manage HCMV infection. The pyrophosphate analog phosphonoformic acid (PFA, foscarnet) provides an option to treat HSV1, HSV2, VZV and HCMV, if first-line drugs have failed to lower the viral burden. Like all current antiviral treatments, long-term treatment can lead to the development of drug resistance. Severe side effects and complicated treatment schedules represent other problems in the management of herpesvirus infection.

Unfortunately, the development of assays to screen for novel anti-herpetic DNA polymerase inhibitors has been limited by technical problems. For the purpose of biochemical screens, *herpesviridae* DNA polymerases are difficult to overexpress in heterologous expression systems and have limited solubility. Hence, it has been difficult to characterize structural and functional details of these polymerases [2,3,4,5,6,7,8]. Of the eight human herpesvirus DNA polymerases, the best-studied is perhaps UL30 from HSV1. This enzyme has been characterized extensively biochemically and has been successfully crystallized [9]. Progress has also been made in characterizing HCMV UL54 [10,11]

In contrast to *herpesviridae* DNA polymerases, the orthologues enzymes of bacteriophage T4 (T4gp43) and “T4 like” bacteriophage RB69 (RB69gp43) are well studied. T4gp43 has been studied extensively using genetic, molecular biology, and biochemistry. Research into T4gp43 has been key to our current understanding of the dynamics of DNA replication [12]. RB69gp43 has been crystallized in various forms and therefore provides an important structural model for polymerases that belong to the same family [13,14,15,16]. It is here attempted to discuss the general aspects of structure and function of these related enzymes and the utility of RB69gp43 as a surrogate system for *herpesviridae* DNA polymerases in efforts to provide a better understanding of mechanisms of drug action and resistance. 

## 2. Structure and Function of B Family Polymerases

**Figure 1:**
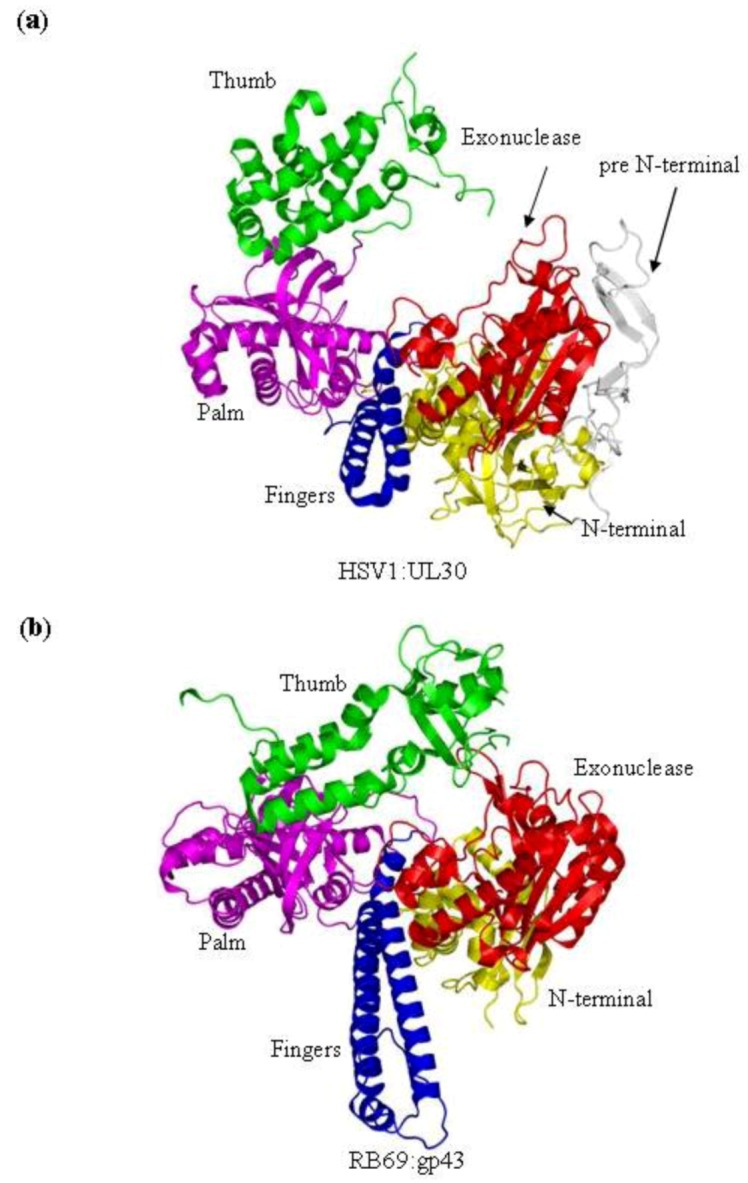
(**a**) Domain structure of HSV1 UL30 (pdb 2GV9) [9]. The pre N-terminal domain is shown in white, the N-terminal domain is yellow, the exonuclease domain is red, the palm domain is magenta, the fingers domain is blue and the thumb domain is green. (**b**) The structure of the RB69gp43 apo form (pdb file 1IH7) [15]. Both structures show the fingers subdomain the open conformation. Images were generated using Pymol [23].

DNA dependant DNA polymerases can be subdivided into five different families based on sequence and structural homology [17]. The DNA polymerases of bacteriophage RB69 and the *herpesviridae* are classified as B family polymerases (Figure 1a,b) [18]. B family polymerases have been identified in all domains of life and are primary involved in genome replication [19]. Unlike other polymerase families, the B family polymerases form part of a multi-subunit complexes, sometime referred to as the DNA replisome, which can co-ordinate both leading and lagging strand replication [17]. However, the polymerase catalytic activity of B family DNA polymerases is encoded by a single gene, which is sometimes referred to as the DNA polymerase catalytic subunit [20]. The catalytic subunit also often encodes an intrinsic 3'–5' exonuclease activity which provides proofreading. This substantially increases the accuracy of DNA synthesis [21,22]. The B family catalytic subunit, in the presence of the polymerase accessory proteins, is both high faithful in replicating DNA and are highly processive [12]. 

**Figure 2:**
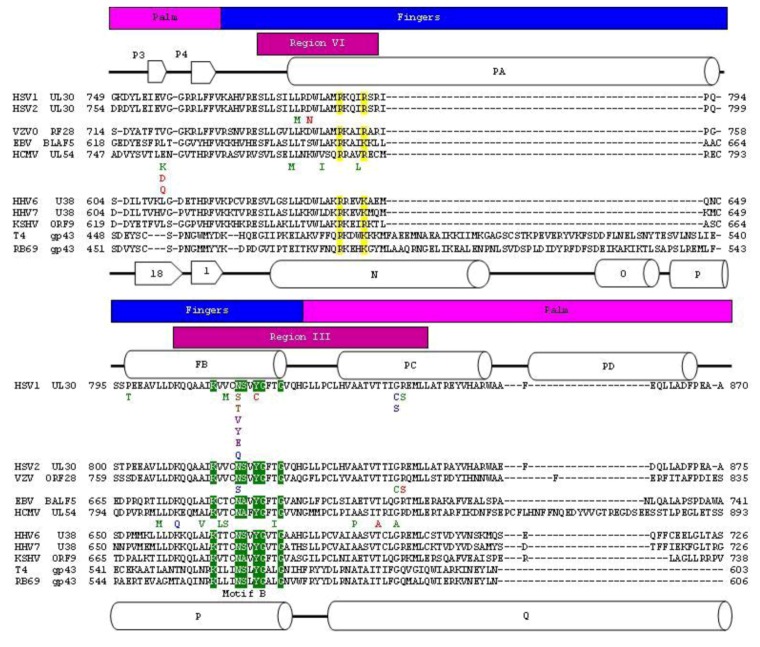
Protein sequence alignment of herpesvirus and bacteriophage polymerase finger domains. Herpesvirus and bacteriophage polymerase were aligned individually using the Muscle algorithm within Geneious [24,25]. The bacteriophage and herpesvirus sequences were then structurally aligned by RAPIDO [26]. Blocks above sequence highlight structural domains of polymerase. The palm domain is in pink. The fingers domain is in blue. The known conserved regions are shown in magenta blocks above sequence [27,28,29,30,31]. Secondary structural elements of HSV1 UL30 are indicated and are number according to Liu *et al.* (2006). Secondary structural elements of RB69 gp43 are indicated and are numbered according to Wang *et al.* (1997). Structural motifs are highlighted in sequence. N helix residues are in yellow. Motif B is in green. Mutations that have been associated with resistance to current anti-herpetic drugs are shown below the corresponding residue [32,33]. Resistance mutations are colored using the following scheme: Red: Pyrophosphate^R^ (Resistant), Blue: Nucleotide^R^, Green: Pyrophosphate^R^ and Nucleotide^R^. Purple: Pyrophosphate^HS ^(Hypersensitive), Brown: Nucleotide^R^ but Pyrophosphate^HS^.

RB69 and each of the members of the *herpesviridae* family encode a B family polymerase (Figure 1 and Figure 2). The virally encoded polymerase serves to replicate the viral genome. Both RB69gp43 [13] and HSV1 UL30 [9] have been studied using X-ray crystallography. Both polymerases are composed of five conserved structural domains, referred to as N-terminal, 3'–5' exonuclease, palm, fingers and thumb subdomains. In addition to these five conserved domains, the x-ray crystal structure of HSV1 UL30 showed an extra domain at the N-terminal end of the protein, which is called the pre N‑terminal domain (Figure 1b).

**Appendix Figure A1:**
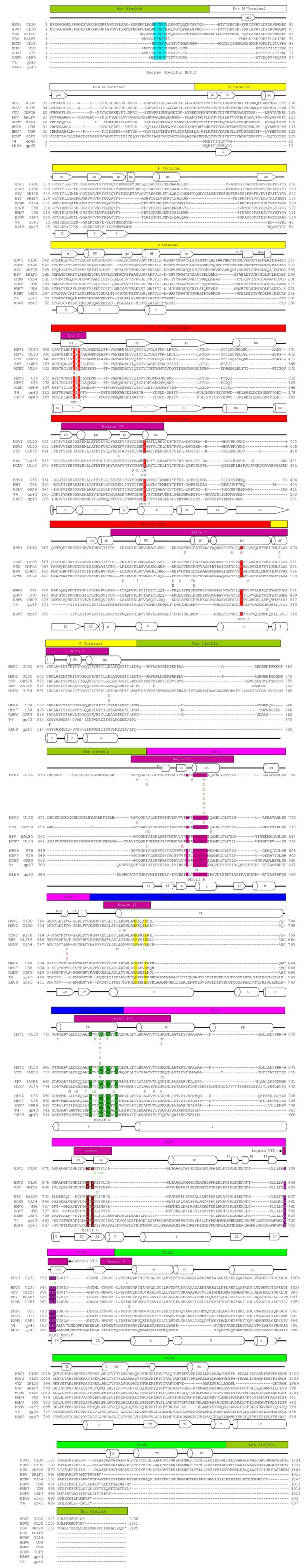
Protein sequence alignment of herpesvirus and bacteriophage polymerases based on structural data. Herpesvirus and bacteriophage polymerase were aligned individually using the muscle algorithm within Geneious [24,25]. Then the bacteriophage and herpesvirus sequences were aligned using an alignment based on a structural alignment of HSV1 UL30 (2GV9) and RB69 gp43 (1IH7) produced by RAPIDO [26]. Blocks above sequence highlight structural domains of polymerase. Regions unresolved in the HSV1 structural model are shown in green. The pre N terminal domain is in white. The N terminal domain is in yellow. The 3'–5' exonuclease domain is in red. The palm domain is in pink. The fingers domain is in blue and thumb domain is in green. The known conserved regions are shown in magenta blocks above sequence [27,28,29,30,31]. Secondary structural elements of HSV1 UL30 are indicated. Elements are number according scheme provided in Liu *et al.* (2006). Secondary Structural elements of RB69 gp43 are indicated. Elements are numbered according scheme provided in Wang *et al.* (1997). Structural motifs involved in polymerase and exonuclease activity are highlighted in sequence. Herpes virus specific motif is in blue. Exonuclease conserved residues are in red. Motif A is in magenta. N helix residues are in yellow. Motif B is in green. Motif C is in brown. KKRY motif is in purple. Mutations that have been associated with resistance to anti-herpetic drugs are shown below the corresponding residue [32,33]. Resistance conferring mutations are colored using the following scheme: Red: Pyrophosphate^R^ (Resistant), Blue: Nucleotide^R^, Green: Pyrophosphate^R^ and Nucleotide^R^. Purple: Pyrophosphate^HS ^(Hypersensitive). Pink: Pyrophosphate^R^ but Nucleotide^HS^, Brown: Nucleotide^R^ but Pyrophosphate^HS^.

Structures of RB69gp43 in various forms in the absence and presence of substrates provided a detailed insight into distinct events involved in nucleotide incorporation, as well as the dynamics of exonuclease function. The structure of the HSV1 UL30 apo enzyme shows a similar domain structure as seen with RB69gp43; however, the exact structural requirements for DNA and nucleotide binding have yet to be established in this case. HSV1 UL30 contains the particular conserved motifs shared by all B family polymerases (Appendix Figure A1). 

## 3. Subdomains of B Family Polymerase

Protein sequence alignments of HSV1 UL30 to other *herpesviridae* DNA polymerase show that it is likely that all Human *herpesviridae* DNA polymerases contain a pre-N terminal domain [9]. The exact function of the pre-N terminal this domain remains elusive. The pre-N terminal domain also contains the FYNPYL motif specific to *herpesviridae* family polymerases (Appendix Figure A1) [9]. It has recently been shown that this motif is required for efficient replication of viral DNA synthesis *in vivo*; a mutant polymerase lacking the FYNPYL motif showed a substantial reduction in viral DNA synthesis [34]. However, the purified FYNPYL deletion mutant showed no reduction in polymerase activity, suggesting that this motif may have a function in the formation of the viral DNA replisome.

The N-terminal domain shows a βαββαβ fold, which has been found in some RNA binding proteins [9,35]. In addition, the crystal structure of the RB69 gp43 contains a rGMP bound to the N‑terminal domain [15], and some mutations in the N-terminal domain of T4gp43 decrease the expression of the polymerase leading to the suggestion that the N-terminal domain may be involved in expression regulation [36]. However, in spite of these observations, the functional role of this domain remains to be defined. 

The sequence of the 3'–5' exonuclease domain of B family polymerases is not highly conserved. However, all 3'–5' exonuclease domains currently characterized adopt a ribonuclease H-like (RNase H‑like) fold. The RNase H fold brings four highly conserved negatively charged residues together to form the active site. In both RB69gp43 and HSV1 UL30 these residues have been identified as three aspartic acids and a glutamic acid. These residues are essential for the binding of two divalent, catalytic metal ions. Structural elements, that harbor active site residues in RB69gp43, are referred to as exoI (D114, E116), exoII (D222), and exoIII (D327) (Table 1, Appendix Figure A1). In *herpesviridae* polymerases the equivalent regions are referred to as ExoI, region IV and Delta (δ) C, respectively. In HSV1 UL30 these residues are; ExoI, D368 and E370, region IV or ExoII, D471 and delta C or ExoIII, D581 (Table 1, Appendix Figure A1). 

**Figure 3:**
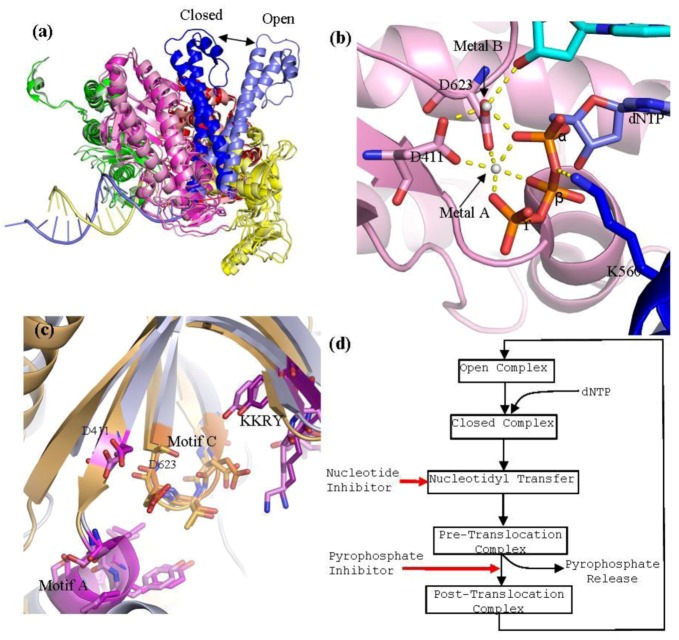
(**a**) Superpositioning of open and closed structures of RB69 gp43 showing finger domain movement. This diagram is composed of RB69 gp43 in the fingers closed position (pdb 3LDS) [37] and in the fingers opened position (pdb 1IH7) [15]. (**b**) Polymerase active site of RB69 gp43 showing interactions between conserved residues of motif A and C, metal ions A and B and dNTP and interactions between K560 and dNTP. This image is an aligned composite image of pdb 3LDS [37] and 3SCX [38]. (**c**) Structural alignment of the polymerase active site of RB69gp43 (pdb 3LDS) and HSV1 UL30 (pdb 2GV9). RB69 gp43 backbone is in light blue while the HSV1 UL30 backbone is in light orange. Active site residues of RB69 gp43 are indicated. RB69 gp43 motif A is in magenta, motif C is in orange and KKRY is in purple. HSV1 UL30 motif A is in pink, motif C is in light orange and KKRY is in light purple. Images were generated using Pymol [23]. (**d**) Generalized diagram of the polymerase catalytic cycle showing steps at which inhibitors can act. Nucleotide inhibitors, once incorporated, prevent further extension of the DNA primer, by inhibiting nucleotidyl transfer. Whereas, pyrophosphate inhibitors mimicking the pyrophosphate leaving group, stabilizing the pre-translocation complex and prevent translocation.

The polymerase active site of B family polymerases is made up of the three domains; the palm, fingers and thumb domains. Together they adopt the classic right hand conformation seen in all available structures of viral polymerases (Figure 1). Two highly conserved motifs in the palm domain, one in the fragment of the palm domain prior to the fingers domain called motif A and one in the fragment post fingers domain called motif C (Table 1, Appendix Figure A1) are likewise seen as signature motifs. In *herpesviridae *polymerases these domains are referred to as region II and region I, respectively. These motifs are; motif A; DXXLYPS and motif C; DTDS (Table 1, Appendix Figure A1). Structurally, these motifs fold together to form a three-strand anti-parallel β sheet (Figure 3c) [13]. Two conserved aspartic acid residues, D411 from motif A and the D625 from motif C, are required to form critical interactions which help co-ordinate the two divalent metal cation that are critical for DNA polymerization (Figure 3b) [15]. In addition, to motif A and C there are two other conserved motifs which also form parts the active site, motif B (KXXXNSXYG), which is known as Region III in *herpesviridae*, which is located on the helix P of the fingers domain, and the KKRY motif which is in the palm domain sequentially after motif C (Appendix Figure A1). 

**Table 1:** Comparison of size, weight and position of conserved motifs or active site residues of RB69 and *herpesviridae* DNA polymerases. The position conserved motifs where assigned based on alignments generated using geneious [24].

Virus (Gene)	Amino Acids (aa)	Weight (kDa)	FYNPYL motif	Exo 1	Exo2/region IV	Exo3/delta (ä) C	Motif A/region II	Motif B/region III	Motif C/region I	KKRY motif
**RB69 (gp43)**	903	104.47	N/A	113–117	222	327	411–420	560–571	621–624	804–807
**HSV1 (UL30)**	1236	136.42	167–173	367–371	471	581	717–726	811–822	886–889	938–941
**HSV2 (UL30)**	1241	137.32	166–172	368–372	472	582	722–731	816–827	891–894	943–946
**VZV (Orf28)**	1195	134.05	7–13	348–352	452	562	682–691	775–786	851–854	903–906
**EBV (BALF5)**	1016	113.43	6–12	295–299	384	497	584–593	681–692	755–758	807–810
**HCMV (UL54)**	1242	137.21	2–8	300–304	413	542	717–726	811–822	910–913	962–965
**HHV6 (UL38)**	1013	115.67	6–12	281–285	369	482	572–581	666–677	740–743	792–795
**HHV7 (UL38)**	1014	115.91	6–12	280–284	368	480	572–581	666–677	740–743	792–795
**KSHV (Orf9)**	1013	113.33	3–9	295–299	383	498	585–594	681–692	752–755	804–807

Residues K560 and N564 of motif B are important in coordinating the tri-phosphate tail of the incoming dNTP during catalysis [15]. Lysine 560 also serves as a proton donor during catalysis (Figure 3b) [39]. Residue Y567 of motif B has been shown to be involved in forming an important interaction with the minor groove of the DNA [40]. This interaction has been shown to be highly important in maintaining polymerase fidelity [40]. In addition to motif B of helix P, there are also conserved positively charged residues within helix N (R482 and K486) that form important interactions with the tri-phosphate tail of the incoming dNTP during catalysis. 

The KKRY motif is primarily involved in stabilizing the B form of DNA. Residue Y708 forms a hydrogen bond with the 3' terminus of the primer while the K705 and R707 form interactions with the phosphate backbone. Together, these interactions help stabilize the interactions between base pairs of the primer and template strands [15]. 

## 4. Catalytic Cycle of B Type Polymerase RB69gp43

Both RB69gp43 and HSV1 UL30 have been crystallized in the so-called open conformation [9,13], but only RB69 has been crystallized in the closed conformation [15]. The closed conformation of a ternary complex contains a DNA primer-template pair and a trapped nucleotide. The most striking difference between the open and closed formation of RB69gp43 is the movement of the fingers domain. Structural alignment of the open and close conformation of RB69gp43 shows that, upon dNTP binding, the fingers domain rotates 60° inwards relative to the palm domain, with the tip of the fingers moving approximately 30 Å (Figure 3a) [15]. Closure of the fingers domain moves the residues on helix N and P that are involved in binding the tri-phosphate tail of the incoming dNTP, 4–8 Å closer to the polymerase active site. This action traps the dNTP in the active site and allows the nucleotidyl transfer to take place. By contrast, the overall structure of the thumb and palm domain between the open and closed conformation remains relatively unchanged. The thumb domain moves approximately 8° toward the palm domain. This action wraps the minor groove of primer-template duplex [15].

B family polymerases employ a two divalent metal ion mechanism for the nucleotidyl transfer [41], in conjunction with a concurrent two proton transfer reaction [39,42]. The catalytic ions are bound by D411 of motif A and D623 of motif C, and form an extensive network of interactions with the dNTP aligning it in the correct orientation for polymerization (Figure 3b). Metal ion A is required to activate the 3'-OH of the primer terminus. Interaction between metal ion A and the primer terminus attract the primer terminus closer to the α-phosphorus atom of the incoming dNTP. This lowers the pKa of the 3'‑OH group allowing it to be deprotonated, which facilitates the nucleophilic attack on the α‑phosphorous atom of the nucleotide substrate [43]. Metal ion B orientates the dNTP triphosphate tail and helps stabilizes the transition states; it has also been suggested that it assists in pyrophosphate release [38]. In RB69gp43, lysine 560 acts as a proton donor [42] and is required to protonates the pyrophosphate leaving group, which may facilitate its release from the complex. This step formally ends the catalysis, leaving the complex now in the pre-translocational state. The fingers of the polymerase rotate away from the active site allowing the release of the pyrophosphate, which allows the DNA substrate to translocate relative to the enzyme. This movement shifts the new 3'-OH terminus into the 1+ position forming a post-tranlocated complex with the polymerase reset for a new catalytic cycle. Polymerases are in general able to discriminate between correct and incorrect nucleotides [44]. Incorrect binding of a nucleotide destabilizes the closed ternary complex, which enables the fingers domain to return to the open form releasing the incorrect nucleotide. Effective discrimination against the incorrect nucleotide at the level of substrate binding raises the fidelity of DNA synthesis significantly [45]. However, in B family polymerases, if an incorrect nucleotide is indeed incorporated, these enzymes can switch into the 3'–5' exonuclease mode and remove the misincorporated base. The 3'–5' exonuclease activity has been shown to increases the fidelity of the RB69 polymerase from the Exo^−^ rate of 2.8 errors per genome (µ_g_) to the Exo^+^ error rate of 4 × 10^−3^ µ_g_ [40,46]. The excision of an incorrectly incorporated terminal nucleotide is also dependant on two divalent metal ions. Structures of RB69gp43 have shown that the exonuclease active site is located approximately 40 Å from the polymerase active site [47]. Thus, for 3'–5' exonuclease activity to occur the DNA primer-template terminus must be translocated from the polymerase active site to the exonuclease site. The details of this process remain to be defined. It has been suggested that DNA replication accessory proteins, particularly the sliding clamp gp45, may be involved in the process of translocating DNA from polymerase to exonuclease active site [15]. It has also been shown that a β hairpin loop between residues 251–262 is important for exonuclease function [48,49]. This loop is involved in stabilizing the frayed base pair at the exonuclease active site allowing the removal of the incorrect nucleotide. During translocation from the polymerisation active site to the exonuclease active site the primer-template pair is partially melted, producing three unpaired bases. The three unpaired bases of the primer strand are then sequestered into the exonuclease active site, which facilitates the excision. Structures of RB69gp43 poised with its primer-template in the exonuclease mode are available [47,48]. 

## 5. Base Selectivity in RB69 DNA Polymerase

A large number of mutant RB69 and T4 DNA polymerases that affect both efficiency and fidelity of DNA replication have been isolated and characterized. In the case of RB69, X-ray crystallography has firmly established the nucleotide binding site at the atomic level. Crucial residues include K560 (Motif B), L415 and Y416 (Motif A) and L561 (Not conserved), Y567 and G568 (Motif B) [50]. 

Conserved residue Motif A has been shown to be important for sugar selectivity. Y416 forms a stacking interaction with deoxyribosyl moiety of the incoming dNTP [15]. It has been proposed that the 2'-OH group of a mismatched rNTP would cause a steric conflict with Y416 preventing formation of a stable complex [51]. Biochemical experiments with the Y416A mutant enzyme corroborated this notion. Unlike the wild type, the mutant was able to incorporate rCTP, ddCTP, and dCTP at similar rates [51].

Residue Y567 of motif B has been shown to be important in maintaining the fidelity of base selection [40,52]. This residue forms an interaction—via a water molecule—with the minor groove of the terminal primer-template pair. This interaction is important for sensing the geometry of the newly form base pair and thus detecting distortions caused by incorrect base pairing [15]. Mutations to residue 567 increase the size of the nascent base-pair-binding pocket allowing the misincorporation of nucleotides. 

**Figure 4:**
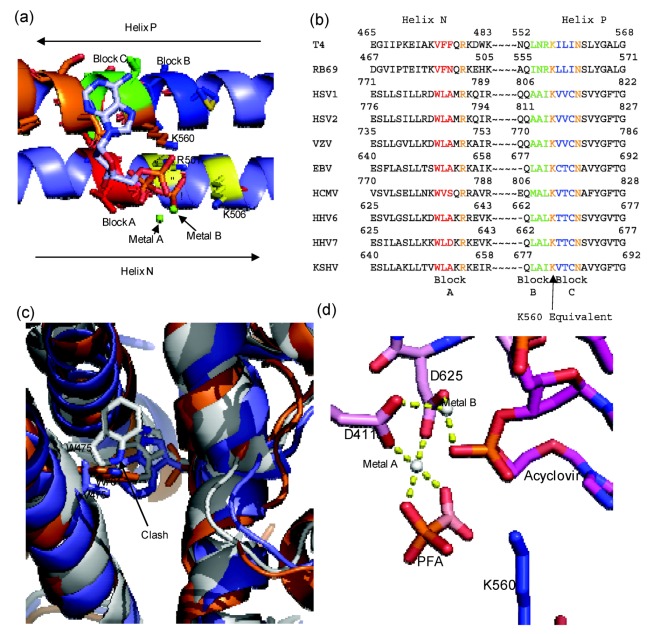
(**a**) Diagram of RB69 Fingers domain showing location of ABC block mutation relative to dNTP binding residues. Motif B residues are in orange, Tri-phosphate interacting residues on Helix N are in yellow, Block A is in red, Block B is in dark blue and Block C is in yellow. Aligned image of pdb 3LDS (dNTP) and pdb 3KD5 (N and P helix). (**b**) Sequence alignment of RB69 and *herpesviridae* sequences showing location of block mutations. (**c**) Diagram showing clash between W478 of Block A and W365. RB69 ABC5 is in white (3KD5), RB69 WT is in blue (pdb 1IH7) and HSV1 UL30 is in orange (pdb 2GV9). (**d**) Diagram of RB69 ABC5 block mutations active site showing phosphonoformic acid binding in β and γ phosphate position and with acyclovir in the pre-translocation position (pdb 3KD5). Images were generated using Pymol [23]. Alignment was generated using Geneious [24].

Residue L561 protrudes into the major groove of the templating base. It has been proposed that this residue is involved in detecting mismatches that lead to distortion of the major groove. L561A mutant confers a mutator phenotype [53]. Interestingly, the equivalent residue in herpesvirus-associated polymerases are not conserved (Figure 4b).

## 6. The Active Site of HSV1 UL30 and RB69gp43

At both the nucleotide and amino acid level, *herpesviridae polymerases* are not highly conserved (Appendix Figure A1). They range in length from 1013–1236 aa, and range in molecular weight from 113 to 137 kDa (Table 1). RB69gp43 is relatively small with 903 aa in length and a molecular weight of 104 kDa (Table 1). In terms of homology, there is very low homology between RB69gp43 and *herpesviridae* DNA polymerases sequences. However, both RB69gp43 and *herpesviridae* contain all the conserved motifs associated with B family polymerases (Appendix Figure A1). 

Unlike herpesvirus DNA polymerases, there is a wealth of information on the structure and function of RB69gp43. Thus, this enzyme is often used as a model for herpesviridae DNA polymerase. Because of the shared conserved motifs between B family polymerase it is reasonable to assume that both herpesviridae and RB69gp43 have a similar, if not an identical catalytic mechanisms. The residues of motif A and C in the HSV1 UL30 and RB69gp43 structure are superimposable, supporting the notion that the function of these residues is similar (Figure 3c). In contrast to the metal cation-binding portion of the active site, the residues of helix N and P of the fingers domain vary greatly between HSV1 UL30 and RB69 gp43. On helix N, the only conserved residues are two basic amino acids R482 and K486 in RB69 gp43, and R785 and R789 in HSV1 UL30. Likewise, on the helix P, the only conserved residues are those of motif B: K560 to N565 in RB69gp43 and K811 to N815 in HSV1 UL30. Thus, the non-conserved residues of helix N and P account for the major differences in the nucleotide binding site, which could in turn account for the difference in sensitivity to antiviral drugs when herpesviridae polymerases are compared with RB69gp43. This notion is supported by biochemical studies with mutant enzymes derived from HCMV UL54 (see below) [11]. 

## 7. Mutations of Bacteriophage T4 that Induce PAA Sensitivity

There are several known natural mutations of bacteriophage T4 DNA polymerase (T4gp43) that affect sensitivity to the pyrophosphate analog phosophonoacetic acid (PAA). T4gp43 is highly homologues to RB69gp43, and, like RB69gp43, is naturally resistant to PAA. However, mutants of bacteriophage T4 with reduced plaque formation in the presence of PAA have been identified [54,55]. One of these mutations, L412M, is located in the conserved motif A. L412M confers increased sensitivity to PAA and interestingly also confers mutator properties to the polymerase [54]. Another interesting feature of the L412M mutant phage is that it can replicate in *E. coli* strains with restricted dGTP pools (*optA1*). The ability to grow under these conditions suggests that the mutant polymerase can make more efficient use of the nucleotide substrates. Because fidelity of DNA synthesis is in part controlled by the rate with which a frayed primer switches from polymerase to exonuclease activity, a bias toward stabilizing the frayed primer in the polymerase active site would cause an overall reduction in enzyme fidelity. When this hypothesis was tested with purified T4gp43 L412M enzyme, it was found that T4gp43 L412M exhibited less exonulease activity in relation to the wild type supporting the notion that the L412M mutation was in fact changing the partitioning between exonuclease and polymerase activity away from exonuclease activity. The equivalent residue to L412 in RB69gp43 is L415. Mutations at this residue show increased rates of mis-incorporation [56]. Interestingly, several suppressor mutants of L412M were also isolated. When subjected to a similar analysis, they were found to be antimutator mutations and unable to grow on *E. coli optA1* [55]. Mutations R335C and S345F are both located within the delta C region of the exonuclease domain (Appendix Figure A1). It has been proposed, that these mutation may affect pyrophosphate sensitivity by increasing the opportunity for exonuclease activity [54]. Since the primer terminus needs to be physically transferred to the exonuclease site, any increase in stability of the primer-terminus in the exonuclease active site or decrease of stability of the primer-terminus in the polymerase active site would increase the disruption of the pyrophosphate analog inhibited complex and henceforth increase the polymerase resistance to inhibition by pyrophosphate analogs. Interestingly in the RB69gp43 crystal structure the equivalent residue to R335 is R338, which is positioned at the very C terminus of the exonuclease domain and points into the cleft in which the finger domain rotates into during catalysis. Thus, the R335 mutation may not directly affect exonuclease function, but may instead inhibit finger domain movement [57]. 

## 8. Chimeric Enzymes

Tchesnokov *et al.*(2009) engineered an RB69gp43-UL54 chimeric enzyme, by mutating the active site of RB69gp43 to include the non-conserved elements from helix N and P of the HCMV enzyme. Swapping the polymerase active sites produced an enzyme that can be expressed in *E. coli*, and is soluble and easily purified. Biochemical assays have shown that the chimeric enzyme is sensitive to the nucleotide inhibitor acyclovir and the pyrophosphate analog PFA [58]. 

Three blocks of non-conserved amino acid residues were considered to engineer the chimera. Block A is located on helix N, and consists of residues 478–480 from RB69gp43 (VFN), these residue were replaced with equivalent residues of HCMV UL54: residues 779 to 781 (WVS). Block B and Block C are both located on Helix P. Block B consist of residues 557–559 of RB69gp43 (INR) and Block C 561–563 (LLI) of RB69gp43 where replaced with residues 808–810 (MAL) and 812–814 (VTC) from HCMV UL54, respectively. Block B and C flank residue K560, which is the conserved basic amino acid that likely donates a proton to the pyrophosphate leaving group [39,42]. Previous studies had already shown that several amino acids within this region can affect sensitivity to PFA (Figure 2) [11]. The chimeric polymerase reproduces drug sensitive and drug resistant phenotypes in cell-free biochemical assays, which validates this enzyme as a model system for polymerase active site inhibitors. 

The structure of the chimera provides a detailed understanding of the mechanism of action of PFA [59]. The enzyme was co-crystallized in complex with a primer-template terminated with acyclovir in the presence and absence of PFA. PFA is bound at the polymerase active site and traps the enzyme in the pre-translocational state. The compound interacts with metal ion B and residue R482 of helix N, similar to the interactions formed by the β- and γ-phosphate of a bound dNTP in the post‑translocational conformation. It appears that W478 of block A is critical in mediating sensitivity to PFA. Although no additional contacts are formed, this residue likely reduces the population of complexes that exist in the open conformation due to steric interference with W365 on helix J. Generally binary complex structures of B family polymerase are found to be in the open conformation; however, in the case of the chimeric enzyme the fingers are in the closed conformation even in the absence of PFA [59]. The predicted steric clash was confirmed with enzymes containing amino acid substitutions at residues 478 and 365 [59]. 

## 9. Resistance to Antiviral Drugs

Resistance-conferring mutations in the polymerase of HSV1, HSV2, VZV and HCMV UL54 have been identified *in vivo* and *in vitro* [10,32,33,60]. Drug resistance is measured as an increase in the inhibitory concentrations of a given drug required to block 50% viral replication (IC_50_). In HSV1, HSV2 and VZV an IC_50_ ≥ 4.4 µM confers significant levels of resistance to acyclovir [32], and in HCMV an IC_50_ ≥ 12µM confers significant levels of resistance to ganciclovir, and IC_50_ ≥ 400 µM to PFA [60]. Because of the difficulties of purifying Herpesvirus DNA polymerase the many resistance mutation have only been assessed in cell-based phenotypic assays [60]. 

All known resistance mutations can be roughly divided into two groups: 1. mutations within the exonuclease domain, which may affect 3'–5' exonuclease function, and 2. mutations within the domains that make up the polymerase active site, which may therefore affect polymerase function directly (Appendix Figure A1). Within the 3'–5' exonuclease domain there are several resistance mutations around the active site residues exo 1, exo2 and exo 3 respectively (Appendix Figure A1). Many of these mutations have been characterized to impair 3'–5' exonuclease activity [61,62]. Interestingly some of these resistance mutations have been characterized to confer resistance to pyrophosphate inhibitors but hypersensitivity to nucleotide inhibitors. An example of this phenotype is the HSV1 mutations Y577H and D581A [62] (Appendix Figure A1). Both mutations are located within the delta C region close to the exo 3 residue. Both mutations have been shown to impair exonuclease activity [62]. Logically a polymerase impaired in 3'–5' exonuclease activity would be unable to remove an incorporated nucleotide inhibitor from viral DNA, which would increase the viruses sensitivity to nucleotide inhibitor, thus explaining the hypersensitivity phenotype. However, the mutations also affect pyrophosphate inhibitors potency and this effect is harder to reconcile. Since pyrophosphate inhibitors mimic the pyrophosphate leaving group, and thus are not incorporated into DNA, the presence or absence of exonuclease activity should not directly affect pyrophosphate analog inhibitors susceptibility. The analysis of T4gp43 PAA sensitive mutants provides some possible insight into a mechanism [54,55]. While working with the T4gp43 L412M mutant they identified several suppressors of PAA sensitivity [54]. These suppressor mutants were shown to be antimutator polymerases, implying that L412M suppressor mutant polymerase were likely to have an altered rate of exonuclease activity compared to the L412M mutants. The authors suggested that because pyrophosphate analogs competitively inhibit polymerase activity by mimicking the pyrophosphate leaving group, that the transition to exonuclease activity could potentially bypass inhibition. Thus, altering the rate of exonuclease activity could potentially affect pyrophosphate inhibitor potency. Unfortunately this hypothesis has not fully been tested in either T4gp43 or herpesviridae DNA polymerases. 

Resistance mutations within the polymerase active site can be arbitrarily split into two groups: 1. Fingers domain mutations and palm domain mutations. In HSV1, HSV2, VZV and HCMV several resistance mutations within the finger domain associated with region VI within helix PA and region III within helix PB. Helix PA and PB are equivalent to helix N and P of RB69gp43 (Figure 2) [32,33]. These two regions make up the finger domain contribution to the polymerase active site. Helix N contains several conserved residues important for nucleotide binding, while Helix P contains conserved residue K560 that is required for proton transfer during catalysis and N564, which is required for nucleotide binding. Crystal structures of RB69gp43 have shown that residue N564 interacts with the β phosphate of the nucleotide via a water molecule [52,63]. There are also several resistance mutations associated with the motif A, C and KKRY within the palm domain (Appendix Figure A1). In Motif A or region II there are several mutations prior to Motif A, which cause a pyrophosphate inhibitor hypersensitive, nucleotide inhibitor resistance phenotype. Mutations in motif C or region I and the KKRY motif or region VII (Appendix Figure A1) have all been characterized as inducing both pyrophosphate and nucleotide inhibitor resistance. Being that motif C and the KKRY motif is involved in aligning the 3'-OH nucleophile during catalysis any subtle change to the positioning of these motifs could change the binding and catalytic constants of the polymerase. 

Several mutations remain to be confirmed as resistance-conferring amino acid substitutions [33]. Because of the difficulties in working with herpesviridae these mutations have not been tested in a defined genetic background to determine the phenotypic effect on resistance or susceptibility. 

## 10. Conclusions

RB69gp43 provides an excellent model for the study of structure–function relationships of B family polymerases. However, there are limitations for the study of orthologous *herpesviridae *polymerases. Most importantly, the phage enzyme is not inhibited by approved drugs that bind to the polymerase active site in either post- or pre-translocational states. Chimeric enzymes composed of a RB69gp43 backbone and important elements of the active site of *herpesviridae *DNA polymerases can potentially address this problem. These findings warrant further investigation in such enzymes as novel tools in future drug discovery and development efforts.
